# H3K23/H3K36 hypoacetylation and HDAC1 up-regulation are associated with adverse consequences in obstructive sleep apnea patients

**DOI:** 10.1038/s41598-021-00052-9

**Published:** 2021-10-19

**Authors:** Yung-Che Chen, Po-Yuan Hsu, Chien-Hung Chin, Chang-Chun Hsiao, Chia-Wei Liou, Ting-Ya Wang, Yong-Yong Lin, Chiu-Ping Lee, Hsin-Ching Lin, Meng-Chih Lin, Mao-Chang Su

**Affiliations:** 1 Division of Pulmonary and Critical Care Medicine, Department of Medicine, Kaohsiung Chang Gung Memorial Hospital and Chang Gung University College of Medicine, 123, Ta-Pei Rd, Niao-Sung District, Kaohsiung City, Taiwan; 2 Sleep Center, Kaohsiung Chang Gung Memorial Hospital and Chang Gung University College of Medicine, Kaohsiung, Taiwan; 3 Department of Medicine, Chang Gung University, Taoyuan, Taiwan; 4 Department of Medical Research, Kaohsiung Chang Gung Memorial Hospital and Chang Gung University College of Medicine, Kaohsiung, Taiwan; 5 Graduate Institute of Clinical Medical Sciences, College of Medicine, Chang Gung University, Taouyan, 33302 Taiwan; 6 Department of Neurology, Kaohsiung Chang Gung Memorial Hospital and Chang Gung University College of Medicine, Kaohsiung, Taiwan; 7 Department of Otolaryngology, Kaohsiung Chang Gung Memorial Hospital and Chang Gung University College of Medicine, Kaohsiung, Taiwan; 8 Chang Gung University of Science and Technology, Chia-yi, Taiwan

**Keywords:** Biomarkers, Diseases, Medical research, Molecular medicine

## Abstract

The aim of this study is to determine the roles of global histone acetylation (Ac)/methylation (me), their modifying enzymes, and gene-specific histone enrichment in obstructive sleep apnea (OSA). Global histone modifications, and their modifying enzyme expressions were assessed in peripheral blood mononuclear cells from 56 patients with OSA and 16 matched subjects with primary snoring (PS). *HIF-1α* gene promoter-specific H3K36Ac enrichment was assessed in another cohort (28 OSA, 8 PS). Both global histone H3K23Ac and H3K36Ac expressions were decreased in OSA patients versus PS subjects. H3K23Ac expressions were further decreased in OSA patients with prevalent hypertension. HDAC1 expressions were higher in OSA patients, especially in those with excessive daytime sleepiness, and reduced after more than 6 months of continuous positive airway pressure treatment. H3K79me3 expression was increased in those with high C-reactive protein levels. Decreased KDM6B protein expressions were noted in those with a high hypoxic load, and associated with a higher risk for incident cardiovascular events or hypertension. *HIF-1α* gene promoter-specific H3K36Ac enrichment was decreased in OSA patients versus PS subjects. In vitro intermittent hypoxia with re-oxygenation stimuli resulted in HDAC1 over-expression and *HIF-1α* gene promoter-specific H3K36Ac under-expression, while HDAC1 inhibitor, SAHA, reversed oxidative stress through inhibiting NOX1. In conclusions, H3K23/H3K36 hypoacetylation is associated with the development of hypertension and disease severity in sleep-disordered breathing patients, probably through up-regulation of HDAC1, while H3K79 hypermethylation is associated with higher risk of cardiovascular diseases, probably through down-regulation of KDM6B.

## Introduction

Affecting approximately 20–30% of men and 10–15% of women, obstructive sleep apnea (OSA) is characterized by chronic intermittent hypoxia with re-oxygenation (IHR) injury, and associated with the increased risks for cardiovascular disease, metabolic syndrome, and all-cause mortality^1–3^. Randomized controlled trials show that continuous positive airway pressure (CPAP) treatment can improve hypertension and insulin resistance, but does not reduce risks of cardiovascular outcomes or death for patients with OSA^4,5^.

In response to external stimuli, such as hypoxia, epigenetic modification can render regulatory elements of genes more or less permissive to interaction with the transcriptional machinery^6^. In our previous epigenome-wide study, we identified and replicated a specific association between a high hypoxic load and *IL1R2* hypomethylation/*AR* hypermethylation, as well as between excessive daytime sleepiness and *NPR2* hypomethylation/*SP140* hypermethylation in the DNA samples from OSA patients^7^. Histone (H) acetylation (Ac) occurs on lysine (K) residues, and promotes relaxation of chromatin structure and transcriptional activation, while histone methylation (me) occurs on lysine (K) and arginine residues on histones H3 and H4, and can be associated with either activation or repression of transcription, depending on the degree (trimethylation (me3); dimethylation (me2); monomethylation (me1)) and location of the modification. Methylation at H3K4, H3K36, H3K79, or H4K20 is associated with transcriptional activation, whereas methylation at H3K9, H3K23, H3K27, and H3K56 is linked with transcriptional repression^8–10^. Histone modifications are mediated by histone-modifying enzymes, including histone acetyltransferases (HAT), histone deacetylases (HDACs), histone methyltransferases, and histone lysine demethylases (KDMs)^11,12^.

Based on previous findings of the relationships between histone modifications and persistent hypoxia^13^, it is hypothesized that OSA patients have aberrant global histone methylation/acetylation patterns and altered expressions of their corresponding enzymes, which may affect disease severity, clinical phenotypes, and outcomes in this chronic IHR-mediated disease. To test this hypothesis, this case–control study checked eleven global histone modification expressions and their corresponding enzymes in the peripheral blood mononuclear cells (PBMCs) from 56 OSA patients and 16 primary snoring (PS) subjects. We correlated these histone markers with the occurrence of prevalent and incident co-morbidities in the following 3 years, and re-checked them in 8 OSA patients who received home CPAP treatment for > 1/2 year (self-reported use for more than 4 h/night)^14^. Furthermore, *hypoxia inducible factor* (*HIF)-1α* and *HIF-2α* promoter specific H3K36Ac and H3K23Ac enrichment was determined in another cohort of 8 PS subjects and 28 treatment-naïve OSA patients, and an in vitro IHR cell culture model was used to verify these molecular changes.

## Results

A total of 16 PS subjects and 56 patients with treatment-naïve OSA were enrolled and analyzed (cohort 1). Demographic, PSG, and blood chemistry data of all the study participants are presented in Table 1. There were no significant differences between two groups in terms of age, gender, BMI, current smoking, alcoholism, co-morbidities, blood cholesterol/triglyceride level, and fasting blood sugar. There were significant differences in PSG parameters and serum hypersensitivity C-reactive protein (hsCRP) levels between two groups.

**Table 1:** Demographic, biochemistry, and sleep data of all the 108 study participants.

	Cohort-1			Cohort-2		
	PS subjects (n = 16)	OSA patients(n = 56)	*p* value	PS subjects (n = 8)	OSA patients (n = 28)	*p* value
Age, years	41.1 ± 12.8	43.3 ± 8.6	0.532	44.8 ± 9.3	50.2 ± 12.4	0.262
Male Sex, n (%)	14 (87.5)	50 (89.3)	0.376	6 (75)	23 (82.1)	0.653
BMI, kg/m^2^	25.2 ± 2.9	25.9 ± 2.8	0.383	24.6 ± 3	26.6 ± 3.1	0.114
AHI, events/hour	3.7 ± 2.5	66.9 ± 12	< 0.001	3.2 ± 2.6	57.2 ± 2.1	< 0.001
ODI, events/hour	2.1 ± 2.2	55.9 ± 20.7	< 0.001	1.2 ± 1.2	45.7 ± 25.9	< 0.001
Mean SaO2, %	96.4 ± 1	93.6 ± 2.4	< 0.001	95.8 ± 0.8	93.7 ± 2.5	0.003
Minimum SaO2, %	86.1 ± 10.1	67.5 ± 14.6	< 0.001	91.2 ± 3.6	70.2 ± 10.7	< 0.001
Snoring index, counts/hour	107 ± 201.5	377.8 ± 197.7	< 0.001	66.4 ± 60.8	358.5 ± 174.3	< 0.001
hs CRP, mg/L	1.79 ± 1.11	3.64 ± 4.73	0.013	NA	NA	
ESS	9.7 ± 4.1	10.6 ± 5.5	0.553	9.6 ± 5.7	10.3 ± 4.9	0.776
EDS, n (%)	4 (21.4)	16 (88.9)	< 0.001	2 (33.3)	10 (41.7)	0.709
Current smoking, n (%)	6 (37.5)	21 (44.7)	0.616	2 (28.6)	9 (32.1)	0.856
Alcoholism, n (%)	0 (0)	3 (6.4)	0.3	0 (0)	2 (7.1)	0.466
Cholesterol, mg/dl	201.5 ± 42.6	196.3 ± 33.5	0.616	180.4 ± 37.6	196.2 ± 42.3	0.442
Triglycerides, mg/dl	166.2 ± 130.7	153.5 ± 86.5	0.566	131.6 ± 137.2	137.2 ± 89.3	0.9
Fasting sugar, mg/dl	100.9 ± 18.8	100.7 ± 11.8	0.944	98 ± 6.4	100.6 ± 25.5	0.823
Hypertension, n (%)	3 (18.8)	18 (35.3)	0.213	2 (25)	9 (32.1)	0.699
DM, n (%)	1 (6.3)	2 (3.9)	0.694	1 (12.5)	4 (14.3)	0.898
Heart disease, n (%)	1 (6.3)	4 (7.8)	0.832	0 (0)	0 (0)	1
Stroke, n (%)	0 (0)	4 (7.8)	0.248	1 (12.5)	0 (0)	0.058
CKD, n (%)	0	1 (1.8)	0.59	0 (0)	0 (0)	1

*PS* primary snoring, *OSA* obstructive Sleep Apnea, *BMI* body mass index, *AHI* apnea hypopnea index, *SaO2* arterial oxyhemoglobin saturation, *ODI* oxygen desaturation index, the number of dips > 4% of basal SaO2%//hour, *hsCRP* hypersensitivity C-reactive protein, *ESS* Epworth Sleepiness Scale, *EDS* excessive daytime sleepiness, *DM* diabetes mellitus, *CKD* chronic kidney disease.

### Differential global histone methylation/acetylation patterns related to OSA and its clinical phenotypes

**Figure 1:**
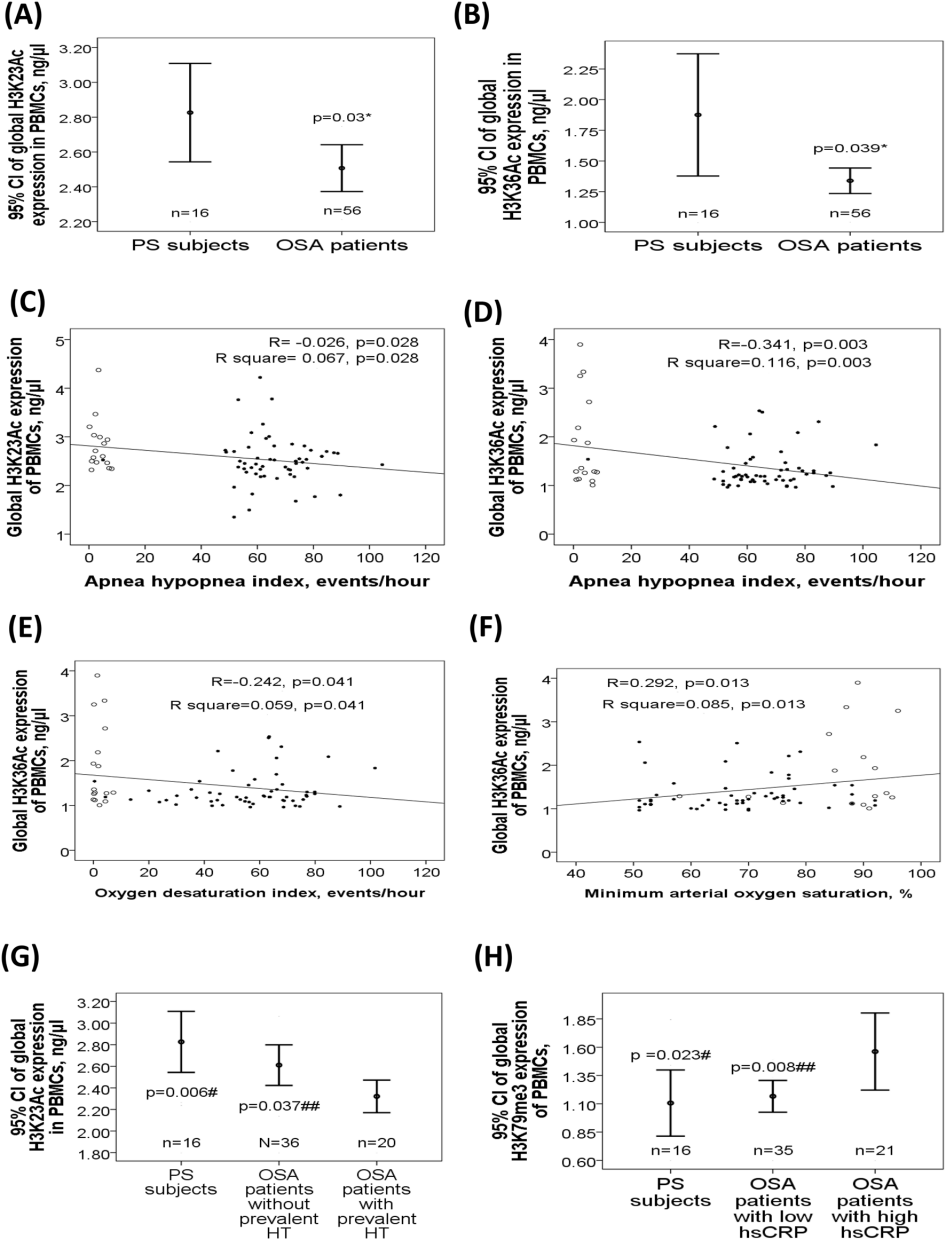
Differential global histone methylation/acetylation patterns related to obstructive sleep apnea (OSA) and its clinical phenotypes. Both (**A**) global histone H3K23Ac and (**B**) H3K36 Ac expressions were decreased in OSA patients. (**C**) H3K23Ac expression was negatively correlated with apnea hypopnea index (AHI). H3K36Ac expression was negatively correlated with both (**D**) AHI and (**E**) oxygen desaturation index (ODI), and positively with (**F**) minimum oxygen saturation (SaO2). (**G**) H3K23Ac expression was further decreased in OSA patients with prevalent hypertension. (**H**) H3K79me3 expression was increased in OSA patients with high serum hypersensitivity C-reactive protein (hsCRP) levels. *adjust for age, body mass index, gender, smoking history, alcoholism history, and co-morbidities (diabetes mellitus, hypertension, stroke, cardiac disease, chronic kidney disease) by multivariate linear regression analysis. #compared between primary snoring (PS) subjects and obstructive sleep apnea (OSA) patients with a particular phenotype (hypertension or high hsCRP), adjusted by multivariate linear regression analysis. ##compared between OSA patients with a particular phenotype (hypertension or high hsCRP) and those without the phenotype, adjusted by multivariate linear regression analysis. Hollow circle indicates PS subjects; solid star indicates OSA patients. PBMC = peripheral blood mononuclear cell; CI = confidence interval; HT = hypertension.

To determine the role of global histone acetylation/methylation in the development of OSA and comorbidities, protein levels of H3K9Ac, H3K14Ac, H3K23Ac, H3K36Ac, H3K56Ac, H4K16Ac, H3K4me3, H3K9me3, H3K27me3, H3K36me3, and H3K79me3 were measured in the PBMC samples from cohort 1. These modification sites were chosen based on their known biological effects on gene expressions and the accessibility of the ELISA kits^8–10^. Both global histone H3K23Ac (2.5 ± 0.51 versus 2.83 ± 0.53 ng/μl, adjusted p = 0.03, Fig. 1A) and H3K36Ac (1.34 ± 0.39 versus 1.88 ± 0.94 ng/μl, adjusted *p* = 0.039, Fig. 1B) expressions were significantly decreased in OSA patients as compared with that in PS subjects, while the other global histone modification expressions were not different between OSA and PS groups (Supplementary Fig. S1). H3K23Ac expression was negatively correlated with both AHI (r = − 0.26, *p* = 0.028, Fig. 1C).The correlation remained statistically significant in multivariate linear regression analysis model 2 (Supplementary Table S1), but did not reach statistical significance in the analysis of separate PS and OSA groups. H3K36Ac expression was negatively correlated with both AHI (r = − 0.341, *p* = 0.003, Fig. 1D) and ODI (r = − 0.242, *p* = 0.041, Fig. 1E), and positively correlated with minimum SaO2 (r = 0.292, *p* = 0.013, Fig. 1F). The three correlations remained statistically significant in multivariate linear regression analyses model 1 or 2 (Supplementary Tables S2, S3, S4), but were reduced in separate PS and OSA groups. Subgroup analysis revealed that global histone H3K23Ac expression was further decreased in OSA patients with prevalent hypertension (n = 20, 2.32 ± 0.32 ng/μl, Fig. 1G) versus those without prevalent hypertension (n = 36, 2.61 ± 0.56 ng/μl, *p* = 0.042/adjusted *p* = 0.037) or PS subjects (*p* = 0.004/adjusted *p* = 0.006). Furthermore, global histone H3K79me3 expression was increased in OSA patients with high serum hsCRP levels (n = 21, hsCRP > 3; 1.56 ± 0.71 ng/μl; Fig. 1H) versus those with low serum hsCRP levels (n = 35, hsCRP≦3; 1.17 ± 0.4 ng/μl, *p* = 0.005/adjusted *p* = 0.008) or PS subjects (1.11 ± 0.51 ng/μl, *p* = 0.048/adjusted *p* = 0.023)^15^.

### Gene and protein expression changes of specific histone modifying enzymes responsible for the global histone methylation/acetylation patterns

**Figure 2:**
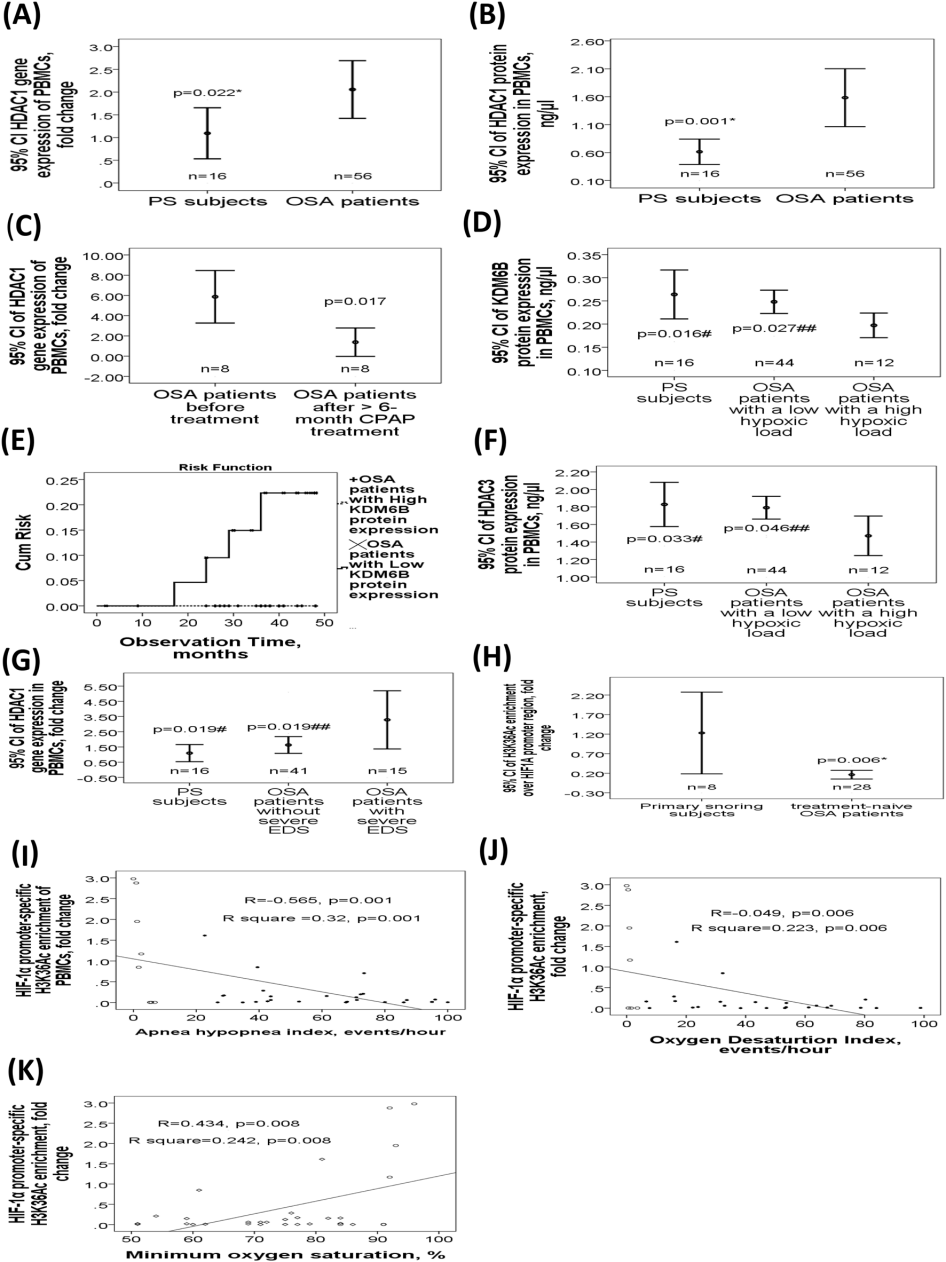
Specific gene and protein expression changes of specific histone modifying enzymes related to OSA and its clinical phenotypes. Both (**A**) *HDAC1* gene and (**B**) HDAC1 protein expressions were higher in OSA patients. (**C**) *HDAC1* gene expression was reduced after more than 6 months of continuous positive airway pressure (CPAP) treatment in 8 selected OSA patients. (**D**) KDM6B protein expression was significantly decreased in OSA patients with high hypoxic load. (**E**) OSA patients with low KDM6B protein expression had a higher risk for recent-onset cardiovascular events or hypertension in the following 3 years than those with high KDM6B protein expression. (**F**) HDAC3 protein expression was decreased in OSA patients with high hypoxic load. (**G**) *HDAC1* gene expression was increased in OSA patients with severe excessive daytime sleepiness (EDS). (**H**) H3K36Ac enrichment over the *HIF-1α* gene promoter region was decreased in OSA patients. This histone marker was negatively correlated with (**I**) apnea hypopnea index (AHI) and (**J**) oxygen desaturation index (ODI), and positively with (**K**) minimum oxygen saturation. *adjust for age, body mass index, gender, smoking history, alcoholism history, and co-morbidities (diabetes mellitus, hypertension, stroke, cardiac disease, chronic kidney disease) by multivariate linear regression analysis. #compared between primary snoring (PS) subjects and obstructive sleep apnea (OSA) patients with a particular phenotype (a high hypoxic load or severe EDS), adjusted by multivariate linear regression analysis. ##compared between OSA patients with a particular phenotype (a high hypoxic load or severe EDS) and those without the phenotype, adjusted by multivariate linear regression analysis. Hollow circle indicates PS subjects; solid star indicates OSA patients. PBMC = peripheral blood mononuclear cell; cum = cumulative; CI = confidence interval.

To determine the role of specific histone modifying enzymes in the development of OSA and comorbidities, gene expression levels of the *KDM1A*, *KDM4B*, *KDM4C*, *KDM5A-D*, *KDM6B*, *DOT1L*, *HDAC1-7*, *KAT2A*, and *KAT6B* genes, and protein levels of the KDM1A, KDM4, KDM5, KDM6B, and HDAC1-4 were measured in the PBMC samples. These enzymes were chosen based on their known biological effects on specific histone modification patterns and the accessibility of the ELISA kits. Both *HDAC1* gene (2.06 ± 2.33 versus 1.09 ± 0.88 fold change, adjusted *p* = 0.022; Fig. 2A) and HDAC1 protein (1.58 ± 1.94 versus 0.61 ± 0.43 ng/μl, adjusted *p* = 0.001; Fig. 2B) expressions were significantly higher in OSA patients than that in PS subjects, while *HDAC1* gene expression was significantly reduced after more than 6 months of CPAP treatment in 8 selected OSA patients (1.38 ± 1.52 versus 5.96 ± 3.08 fold change, *p* = 0.017; Fig. 2C). There was no significant difference of gene or protein expression in the other histone modifying enzymes between two groups (Supplementary Figs. S2, S3 and S4). Subgroup analysis showed that KDM6B protein expression was decreased in OSA patients with a high hypoxic load (n = 12, ODI > 70 events/hour; 0.2 ± 0.04 ng/μl; Fig. 2D) versus those without a high hypoxic load (n = 44; 0.25 ± 0.08 ng/μl, *p* = 0.017/adjusted *p* = 0.027) or PS subjects (0.26 ± 0.1 ng/μl, *p* = 0.036/adjusted *p* = 0.016). OSA patients with low KDM6B protein expression (< 0.21 ng/μl, n = 23) had higher risk for incident cardiovascular events or hypertension in the following 3 years (*p* = 0.014 by Log-Rank test, Fig. 2E) than those with high KDM6B protein expression (≧0.21 ng/μl, n = 33). HDAC3 protein expression was decreased in OSA patients with a high hypoxic load (1.5 ± 0.35 ng/μl; Fig. 2F) versus those without a high hypoxic load (1.8 ± 0.43 ng/μl, *p* = 0.024/adjusted *p* = 0.046) or PS subjects (1.83 ± 0.47 ng/μl, *p* = 0.031/adjusted *p* = 0.033). *HDAC1* gene expression was increased in OSA patients with severe excessive daytime sleepiness (EDS) (n = 15, ESS > 14; 3.28 ± 3.31 fold change; Fig. 2G) versus those without severe EDS (n = 41; 1.63 ± 1.72 fold change, *p* = 0.014/adjusted *p* = 0.019) or PS subjects (1.09 ± 0.88 fold change, *p* = 0.01/adjusted *p* = 0.019).

### Decreased *HIF-1α* gene promoter-specific H3K36Ac enrichment in treatment-naïve OSA patients of cohort 2

Based on the findings that global H3K23Ac and global H3K36Ac were decreased in patients with OSA, we next investigated whether such hypoacetylation occurred specifically at the promoter regions of the *HIF-1α* and *HIF-2α* genes. We examined this possibility by ChIP method in PBMC lysates from another cohort (cohort 2) of 28 treatment-naïve OSA patients, and 8 matched PS subjects (Table 1). H3K36Ac enrichment over the *HIF-1α* (0.52 ± 0.92 versus 1.23 ± 1.25 fold change, adjusted *p* = 0.006; Fig. 2H) gene promoter region was decreased in the treatment-naïve OSA patients as compared with that in the PS subjects. This specific histone modification was negatively correlated with both AHI (r = − 0.548, *p* = 0.001, Fig. 2I) and ODI (r = − 0.449, *p* = 0.006, Fig. 2J), and positively with minimum SaO2 (r = 0.434, *p* = 0.008, Fig. 2K). These correlations remained statistically significant in multivariate linear regression model 1 or model 2 (Supplementary Tables S5, S6, S7), but did not reach statistical significance in the analysis of separate PS and OSA groups. Additionally, H3K36 Ac enrichment over the HIF-2α gene promoter region (0.49 ± 0.3 versus 1.03 ± 1.06 fold change, adjusted *p* = 0.024, Supplementary Fig. S4) was decreased in the treatment-naïve OSA patients versus the PS subjects. However, this difference was not normally distributed and did not reach statistical significance in the non-parametric analysis. Moreover, no significant difference in either HIF-1α or HIF-2α gene expression level was noted between case and control groups (Supplementary Fig. S4).

### Effects of in vitro IHR on histone modifying enzyme expressions and gene-specific histone modifications in human monocytic THP-1 cells

**Figure 3:**
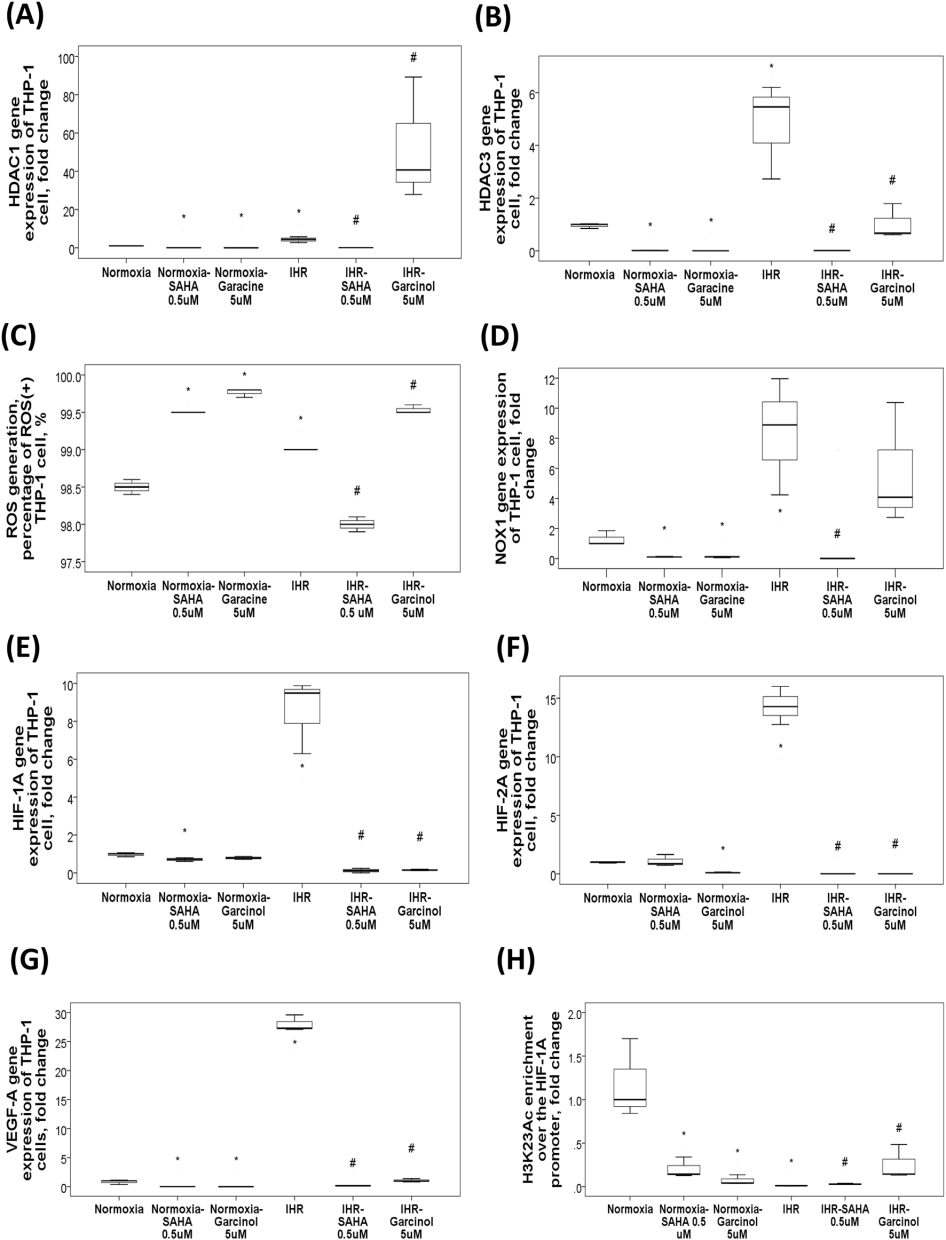
Changes in histone modifying enzyme expressions and H3K23Ac/H3K36Ac enrichment in response to in vitro intermittent hypoxia with re-oxygenation (IHR) stimuli, and the effect of HDAC1 inhibition. HDAC inhibitor, SAHA, treatment resulted in reversion of IHR-induced up-regulations of the (**A**) *HDAC1*, and (**B**) *HDAC3* genes. SAHA treatment reversed (**C**) IHR-induced over-production of reactive oxygen species (ROS) along with (**D**) reversion of NADPH oxidase 1 (NOX1) over-expression. Both SAHA (a HDAC1 inhibitor) and garcinol (a HAT inhibitor) treatment reversed IHR-induced up-regulations of the (**E**) *HIF-1α*, (**F**) *HIF-2α*, and (**G**) *VEGF-A* genes, and (**H**) reversed IHR-induced H3K23 hypoacetylation over the *HIF-1α* promoter region. *p < 0.05, compared with normoxic conditions by U or Kruskal–Wallis test. **p < 0.01, compared with normoxic condition by U-test. #*p* < 0.05, compared with IHR condition by Kruskal–Wallis test. HDAC = histone deacetylase; SAHA = suberoylanilide hydroxamic acid; HAT = histone acetyltransferase; HIF = hypoxia inducible factor; VEGF = vascular endothelial growth factor.

To test the effects of HDAC1 over-expression or under-expression on oxidative stress, THP-1 cells were treated with either suberoylanilide hydroxamic acid (SAHA, HDAC1 inhibitor) or garcinol (HAT inhibitor) under IHR stimuli. Both HDAC1 (Fig. 3A) and HDAC3 (Fig. 3B) gene expressions were increased with 9-day IHR stimuli, while SAHA 0.5 μM treatment resulted in their down-regulations and garcinol 5 μM treatment resulted in their up-regulations under IHR stimuli as compared with IHR alone condition. SAHA reversed IHR-induced elevation of reactive oxygen species (ROS) production (Fig. 3C) in association with normalization of IHR-induced over-expression of the *NOX1* gene (Fig. 3D), while garcinol had the opposite effect. Additionally, SAHA reversed IHR-induced up-regulations of the *HIF-1α* (Fig. 3E), *HIF-2α* (Fig. 3F), and *VEGF-A* (Fig. 3G) gene expressions, and IHR-induced H3K23 hypoacetylation over the *HIF-1α* promoter region (Fig. 3H).

## Discussion

Epigenetic changes are involved in the switching 'on' and 'off' of many genes, and histone modifications has been regarded as epigenetic gene targets for the regulation of disease-associated cellular changes in hypoxic microenvironment. Little is known about the role of histone modifications in OSA, which is characterized by chronic IHR rather than persistent hypoxia. Therefore, in this study, we sought to identify specific histone modification patterns associated with OSA and its clinical phenotypes. We have proved, for the first time, the relationship between global H3K23/H3K36 hypoacetylation in association with *HDAC1* up-regulation and the development of EDS/hypertension phenotype in OSA patients. Specifically, *HIF-1α* gene promoter-specific H3K36 enrichment was decreased in OSA patients and in response to in vitro IHR stimuli. In contrast, global H3K79 hypermethylation along with *KDM6B* down-regulation was found to be related to higher incident cardiovascular risk. Additionally, we found that SAHA, a HDAC inhibitor, could reverse IHR-induced ROS over-production through NOX1 in vitro.

H3K36Ac is localized predominantly to the promoters of RNA polymerase II-transcribed genes, a pattern inversely related to that of H3K36 me^16^. H3K36 acetylation has been shown to increase chromatin accessibility, and promotes DNA double-strand break repair. Furthermore, H3K36 modification is cell cycle regulated with chromatin-bound H3K36me3 peaking in G1 where non-homologous end joining occurs, while H3K36Ac peaking in S/G2 phase when homologous recombination predominates^17^. The H3K36 hypoacetylation in OSA patients and in response to in vitro short-term IHR stimuli suggest that DNA double-strand break repair may be dampened by chronic IHR in OSA through H3K36 hypoacetylation. The decreased enrichment of H3K36 acetylation over the *HIF-1α* gene promoter region in OSA patients and in response to IHR stimuli suggest that H3K36Ac may play a specific role in regulating *HIF-1α*-mediated adaptive responses to hypoxia. However, separate cohorts were used for the global histone and *HIF-1α* promoter-specific histone enrichment analyses, and this may limit the cause-effect interpretation of the experiments. Moreover, correlations between global H3K23Ac/H3K36Ac expression and metrics such as AHI, arousal index, mean SaO2 were quite weak, so further studies are needed. Finally, many environmental exposures, such as cigarette smoking, may cause specific histone modification change. In the lung tissues of smokers with COPD, the expressions of HDAC2, HDAC5, HDAC8, HDAC7, and HDAC10 were decreased significantly, while H3K23Ac or H3K36Ac has not yet been linked to smoking exposure^18^. However, we applied multivariate linear regression models to adjust for all possible confounding factors.

In line with our findings that global H3K23 was hypoacetylated in OSA patients, H3K23 acetylation has been reported to be reduced in diabetic mice, and its hypo-acetylation contributed to Drosophila learning defect and depression-like behaviors in rats^19–21^. Since the co-existence of both H4K16ac and H3K23ac was correlated with higher gene expression levels in a plant study, and lipopolysaccharide can trigger IL-6 production via enhancing recruitment of H3K23 acetylation to IL-6 promoter region, we speculated that the simultaneous hypoacetylation of H3K36 and H3K23 in OSA patients may lead to impaired DNA repair ability under chronic IHR circumstances, and affect specific adaptive gene responses through regulating *HIF-1α*^22,23^.

Up-regulation of HDAC1 has been shown to be associated with adverse prognosis of various cancers, and plays critical roles in cellular senescence, aging, myelination, and adult neurogenesis^24^. In line with our findings that *HDAC1* was up-regulated in OSA patients and in response to IHR stimuli, HDAC1 inhibition was reported to protect against hypoxia-induced swelling in cardiomyocytes through enhancing cell stiffness^25^. Class I HDACs, such as HDAC1 and 3, can enhance HIF-1α stability and *HIF-1* transactivation function in persistent hypoxic conditions through binding to the oxygen-dependent degradation domain of HIF-1α^26^. Although HDAC1 expressions were reduced after CPAP treatment in a small group of OSA patients, the lack of a time control group or sham CPAP device and the lack of CPAP adherence data limit the interpretation of these data. In line with our findings that *HDAC3* was down-regulated in OSA patients with high hypoxic load, HDAC3 has been reported to mediate cardioprotection of remifentanil post-conditioning by targeting GSK-3beta in cardiomyocytes in IHR injury^27,28^. In contrast, HDAC3 could contribute to IHR-induced cell apoptosis of myocardioblasts^28^. However, the reversion of IHR-induced ROS over-production with SAHA through inhibition of the NOX1 gene indicates that HDAC1/3 inhibition may be a new therapy to reduce oxidative stress in OSA. Several recent studies have shown that HDAC inhibition with SAHA, trichostatin A, or valproic acid, reduced ROS production in experimental diabetes, pulmonary hypertension, and atherosclerosis, through inhibitions of NOX1/2/4/5 expression via epigenetically regulating chromatin accessibility^29–32^. HDAC inhibitors have displayed clinical efficacy in treating specific tumors^33^. SAHA inhibits both class I and class II HDACs, but selectively alters transcription of as few as 2–5% of genes^34^. Further research is required to determine whether HDAC1 inhibition could be used to reduce cardiovascular risk in OSA patients.

H3K79 methylation has been shown to be implicated in several processes of DNA replication and repair of DNA damage^35^. Moreover, H3K79 methylation, shows a high positive correlation with transcriptional activity^36,37^. In this study, the global H3K79 hypermethylation in OSA patients with high hsCRP, which has been linked with a high cardiovascular risk^15^, suggest that H3K79me3 may serve as an endogenous protective mechanism against chronic IHR-induced DNA damage in OSA. Most KDM enzymes are structurally similar to the HIF hydroxylase factor inhibiting HIF-1, suggesting that KDM enzymes may act as molecular oxygen sensors in the cell^38^. Hypoxia-inducible KDMs have been shown to support the adaptive and adverse gene transcription induced by HIF-1α, and they can also control genome-wide chromatin landscape, especially KDMs which demethylate H3K9 and H3K27 sites^39^. The down-regulation of KDM6B in OSA patients with high hypoxic load and incident cardiovascular events in this study suggest that long-term oxidative stress-related insults may impair the maintenance of chromatin landscape and provoke cellular senescence and endothelial dysfunction associated with chronic IHR circumstances in OSA. Although previous studies showed that hypoxia either inhibits or induces the enzymatic activity of KMD6B depending on HIF-2α, KDM6B protein levels remained unchanged under hypoxic conditions and decreased upon re-oxygenation^40–42^. Further study is required to determine whether KDM6B supplementation can reverse H3K79 hypermethylation and ameliorate adverse consequences in severe OSA. One limitation is that this study was conducted using PBMCs; thus, the findings associating KDM6B to future cardiovascular events may not extend to other cell types. Given that a recent U.S. healthcare system–based analysis of mortality and markers of severe morbidity identify sleep apnea as a risk factor for COVID-19 mortality, our results may provide particular insight into immune function with OSA and discover mechanistic pathways underlying COVID-19 morbidity^43^. Another limitation is that we did not check some histone modifying enzymes known to regulate histone methylation, such as other JmjC-KDM demethylases and G9a methylase^44,45^.

In conclusions, H3K23/H3K36 hypoacetylation is associated with the development of prevalent hypertension or disease severity in patients with sleep-disordered breathing, probably through up-regulation of HDAC1, while H3K79 hypermethylation is associated with higher risk of incident cardiovascular diseases, probably through down-regulation of KDM6B. *HIF-1α* and HIF-2α gene promoter-specific H3K36 hypoacetylation may play a role in regulating adaptive gene responses to chronic IHR injury of OSA. HDAC1 inhibition can reduce oxidative stress induced by IHR through inhibition of NOX1. These findings provide further insights into novel epigenetic mechanisms by which chronic IHR leads to adverse consequences in OSA, and opens the possibility of using HDAC1 inhibitor in preventing the morbidities and mortality.

## Methods

### Subjects

This study was approved by the Institutional Review Board of Chang Gung Memorial Hospital, Taiwan (certificate number: 102-3887B). The study participants were recruited from the sleep center of Kaohsiung Chang Gung Memorial Hospital from January 2014 through December 2017. Written informed consent was obtained from each subject participating in the study aged 20 years or older. All the methods were carried out in accordance with relevant guidelines and regulations. The exclusion criteria included ongoing infections, autoimmune disease, use of immunosuppressive agent in the past 6 months, narcolepsy, severe obesity (body mass index, BMI, ≧35 kg/m^2^), old age (> 65 year-old), and those with a BMI < 21 kg/m^2^. OSA (apnea hypopnea index, AHI, ≧5 events/hour) and PS (AHI < 5 events/hour) were diagnosed by full night polysomnography examination at the sleep center of Kaohsiung Chang Gung Memorial Hospital^46^. The Epworth Sleepiness Scale (ESS) recorded at the examination was used to measure sleep propensity in every study subject^47^. Prevalent hypertension was defined as systolic blood pressure ≧140 mmHg and/or diastolic blood pressure ≧90 mmHg or medication use at diagnosis of OSA. Incident cardiovascular events (coronary artery disease: angioplasty, coronary artery bypass grafting, myocardial infarction, or angina) or hypertension were defined as those occurred in the following 3 years after the first blood sampling at diagnosis^48^.

### Isolation of PBMC RNA and protein from whole blood samples

PBMCs were isolated from heparinized blood of all study subjects using a two-layer Ficoll-Histopaque density gradient centrifugation (Histopaque 1.077 and 1.119; Sigma Diagnostics, St.Louis, MO) method. Samples were stored in RNA*later*® RNA Stabilization Solution (Ambion®) at − 80 °C until analysis. An RNeasy®Plus Mini Kit (Qiagen, Hilden, Germany) was used for isolation of high quality total RNA.

### Measurement of histone modifying enzyme gene expressions in the PBMCs using quantitative reverse-transcriptase polymerase chain reaction (RT-PCR)

To determine the expressions of *KDM1A, KDM4B, KDM4C, KDM5A-D, KDM6B, DOT1L, HDAC1-7, KAT2A, and KAT6B* of the isolated PBMCs, the gene expressions were analyzed using quantitative RT-PCR. The house keeping gene *GAPDH* was chosen as an endogenous control to normalize the expression data for each gene. All PCR primers (random hexamers) were designed and purchased from Roche according to the company’s protocols (www.roche-applied-science.com), and their sequences are given in Table 2. A total of 300 ng RNA was used for synthesis of first strand cDNA with QuantiTectReverse Transcription Kit (QIAGEN, Germany), and the reverse transcription reaction was added to 5 μl of master mix (QIAGEN, SYBR Green PCR kit; Roche, Germany). The PCR reactions with 45 cycles of amplification were run in a Roche LightCycle 480 QuantiFast R machine. Relative expression levels were calculated using the ΔΔCt method with the median value for the control group as the calibrator^49^.

**Table 2:** Primer sequences for quantitative real-time polymerase chain reactions used in the present study.

Gene name	Primer sequences	
*KDM1A (LSD1)*	Forward	5′-CATCATGGTGCAAGAAGAGC
	Reverse	5′-ATGTGGGAAAGGCAGACAAG
KDM4B (*JMJD2B*)	Forward	5′-CTGAAATGCGTGTACTGCCG
	Reverse	5′-CGTTGCGGTTCTTGGTGATG
KDM4C (*JMJD2C*)	Forward	5′-GCTCGATTTTCCACAGCCTC
	Reverse	5′-CAGGGTCGGCCACATATTCA
KDM5B (*JAR1D1B*)	Forward	5′-GTACTGTGAAGGACGCACCA
	Reverse	5′-TAGCACCGTTTACAGGCTGG
*KDM6B (JMJD3)*	Forward	5′-CACCCACTGTGGTCTGTTGT
	Reverse	5′-TGTCTCCGCCTCAGTAACAG
*HDAC1*	Forward	5′-CGGTGCTGGACATATGAGAC
	Reverse	5′-TGGTCCAAAGTATTCAAAGTAGTCA
*HDAC2*	Forward	5′-CCAGATGTTCTGGCATCCTC
	Reverse	5′-ACAGCCCCTGTTGTCCTGT
*HDAC3*	Forward	5′-TGGCATTGACCCATAGCCTG
	Reverse	5′-TGCATATTGGTGGGGCTGAC
*HDAC4*	Forward	5′-CGGAAGCATGTGTTTCTGCC
	Reverse	5′-TTCTCCATGGAACGGACAGC
*HDAC5*	Forward	5′-TCTTGTCGAAGTCAAAGGAGC
	Reverse	5′-GAGGGGAACTCTGGTCCAAAG
*HDAC6*	Forward	5′-TGGCTATTGCATGTTCAACCA
	Reverse	5′-GTCGAAGGTGAACTGTGTTCCT
*HDAC7*	Forward	5′-GAACAGTCCATCCCAACAGC
	Reverse	5′-GGCTCCTTCCGTCTCCAG
*HIF-1α* promoter	Forward	5′-CACCCCCATCTCCTTTCTCT-3′
	Reverse	5′-GGGTTCCTCGAGATCCAATG-3′
*HIF-2α* promoter	Forward	5′-GAAGTGCGGAGGCAGGAG-3′
	Reverse	5′-GGCAGCCCACTTTAAAAACTC-3′
*GAPDH*	Forward	5′-GAAGAGCCAAGGACAGGTAC
	Reverse	5′-CAACTTCATCCACGTTCACC

*KDM* Lysine de-methylase, *HDAC* Histone de-acetylase, *LSD1* Lysine-specific demethylase 1, *JMJD* Jumonji domain-containing proteins, *HIF* hypoxia-inducible factor, *GAPDH* Glyceraldehyde 3-phosphate dehydrogenase.

### Measurement of global histone modification patterns and histone modifying enzyme protein expressions in the PBMC samples using ELISA method

Protein levels of H3K9Ac, H3K14Ac, H3K23Ac, H3K36Ac, H3K56Ac, H4K16Ac, H3K4me3, H3K9me3, H3K27me3, H3K36me3, H3K79me3*,* KDM1A, KDM4, KDM5,KDM6B, and HDAC1-4 in the PBMCs were assessed by a commercial ELISA method (Epigentek, USA, and R&D systems, Minneapolis, MN), as described previously^50^. Briefly, 40 μl dye (Bio-Rad Protein Assay Dye Reagent Concentrate #500-0006) was added to 10 μl bovine serum albumin with serial dilution to generate a standard curve for total protein concentration by measuring values at OD 595 nm. Accordingly, protein lysate equivalent to 20 ng of total protein was used for each protocol in ELISA analysis.

### Measurement of gene promoter-specific H3K23Ac and H3K36Ac enrichment with Chromatin immunoprecipitation (ChIP) followed by quantitative RT-PCR

3–5 × 10^5^ PBMCs from another cohort of 8 PS subjects, and 28 treatment-naïve OSA patients were harvested and fixed with 1% formaldehyde at room temperature, followed by glycine to stop the crossing linking reaction. Cells were re-suspended in cell lysis buffer containing 1× Protease Inhibitor Cocktail II (200×, Millipore, USA). Extracts were sonicated using a Standard Sonicator (Diagenode Bioruptor) to achieve chromosome fragment lengths of 200–400 bp. 100 μl sonicated cell extract were diluted in 400 μl ChIP Dilution Buffer (EZ-Magna ChIP^TM^ A/G kit, Millipore, USA), and incubated with 1 μg of anti-H3K23Ac or anti-H3K36Ac (Millipore, USA) at 4 °C for 1 hour while rotating. Two aliquots were reserved as ‘‘controls’’—one incubated without antibody and the other with non-immune Mouse IgG (25 μg, Millipore, USA). Protein A/G magnetic beads were added and incubated at 4 °C overnight while rotating. Cross-linking of the immunoprecipitated chromatin complexes and ‘‘input controls’’ (10% of the total soluble chromatin) were reversed by heating the samples at 62 °C for 4 hours, followed by Proteinase K 1 µL incubation (Millipore, USA). The DNA were purified by spin column and stored at − 20 °C^51^.

For real-time RT-PCR analysis, amplification of *HIF-1α* and *HIF-2α* promoter DNA was performed using the TaqMan reagent kit (PerkinElmer Life Sciences)^52^. The PCR primers for the two selected gene promoters located from − 300 to + 30 bp of their transcription start site were listed in Table 2. The conditions for the reaction were: 50 °C, 2 min; 94 °C, 10 min, then 95 °C, 20 s; 60 °C, 30 s for 50 cycles. Resulting PCR products were measured and elaborated by the sequence detector ABI GeneAmp PCR System 9700 (PerkinElmer Biosystems). Histone enrichment was compared by fold enrichment method, in which the ChIP signals are divided by the no-antibody signals, representing the ChIP signal as the fold increase in signal relative to the background signal^53,54^.

### In vitro human monocyte cell line culture under IHR stimuli

The human monocytic leukemia cell line THP-1 cells obtained from ATCC (1 × 10^6^ cells/ml) were re-suspended in a 5 cm culture dish containing 5 ml RPMI 1640 medium, and then exposed to normoxia or IHR in a custom-designed, incubation chambers which are attached to an external O2-CO2-N2 hand-driven controller as previously described^55^. Air-phase set point for IHR consisted of a 25-min hypoxic period (0% O2 and 5% CO2), followed by 35 min of re-oxygenation (21% O2 and 5% CO2), 7 h each day for 9 days^56^. Control cells were maintained in NOX condition for the same durations. HDAC inhibitor, SAHA (Sigma-Aldrich, Saint Luis, MO, USA), and HAT inhibitor, Garcinol (Sigma-Aldrich, Saint Luis, MO, USA), were used to inhibit or augment HDAC expression, respectively. H2DCFDA (catalog no. D6883; Sigma, USA) was used to measure THP-1 intracellular ROS. Cell-associated mean fluorescent intensity was measured by flow cytometry in FL1 channel at excitation and emission wavelengths of 488 and 535 nm, respectively, using the CytomicsTM FC500 (Beckman Coulter ; USA ). Gene expression levels of the NADPH oxidase NOX1/2/4/5 genes were determined using quantitative RT-PCR method.

### Statistical analysis

Continuous values were expressed as mean ± standard deviation (SD). The differences between two groups were analyzed using the Student’s t-test, Mann–Whitney U-test, or X^2^-test, as appropriate. Subgroup comparisons of continuous variables in the validation cohort were performed using the one-way ANOVA with LSD method for post-hoc tests, followed by multivariate linear regression analysis to adjust for all potential confounding factors (age, BMI, gender, smoking, alcoholism, and co-morbidities), and to obtain adjusted p values. The null hypothesis was rejected at *p* < 0.05. All analyses were performed using SPSS software version 20.0 (SPSS Corp., Chicago).

### Ethics approval and consent to participate

This study was approved by the Institutional Review Board of Chang Gung Memorial Hospital, Taiwan (Certificate number: 102-3887B). Written informed consent was obtained from each subject participating in the study.

## Supplementary Information

Supplementary Information.

## Data Availability

The data that support the findings of this study are available in supplementary information online and from the corresponding author upon reasonable request.

## Abbreviations

OSA: Obstructive sleep apnea
IHR: Intermittent hypoxia with re-oxygenation
CPAP: Continuous positive airway pressure
H: Histone
Ac: Acetylation
me: Methylation
HDAC: Histone deacetylase
KDM: Histone lysine demethylase
PBMCs: Peripheral blood mononuclear cells
PS: Primary snoring
HIF: Hypoxia-inducible factor
BMI: Body mass index
AHI: Apnea hypopnea index
ODI: Oxygen desaturation index
ESS: Epworth Sleepiness Scale
RT-PCR: Reverse-transcriptase polymerase chain reaction
ChIP: Chromatin immunoprecipitation
