# Supplementary material for: H3K23/H3K36 hypoacetylation and HDAC1 up-regulation are associated with adverse consequences in obstructive sleep apnea patients
Source: Sci Rep. 2021 Oct 19;11:20697. doi: 10.1038/s41598-021-00052-9 (PMC8526826; doi:10.1038/s41598-021-00052-9)
Supplement: Supplementary file 1 — Supplementary Information. [file 41598_2021_52_MOESM1_ESM.docx]

**H3K23/H3K36 hypoacetylation and HDAC1 up-regulation are associated with adverse consequences in obstructive sleep apnea patients**

**Supplementary Information**

**
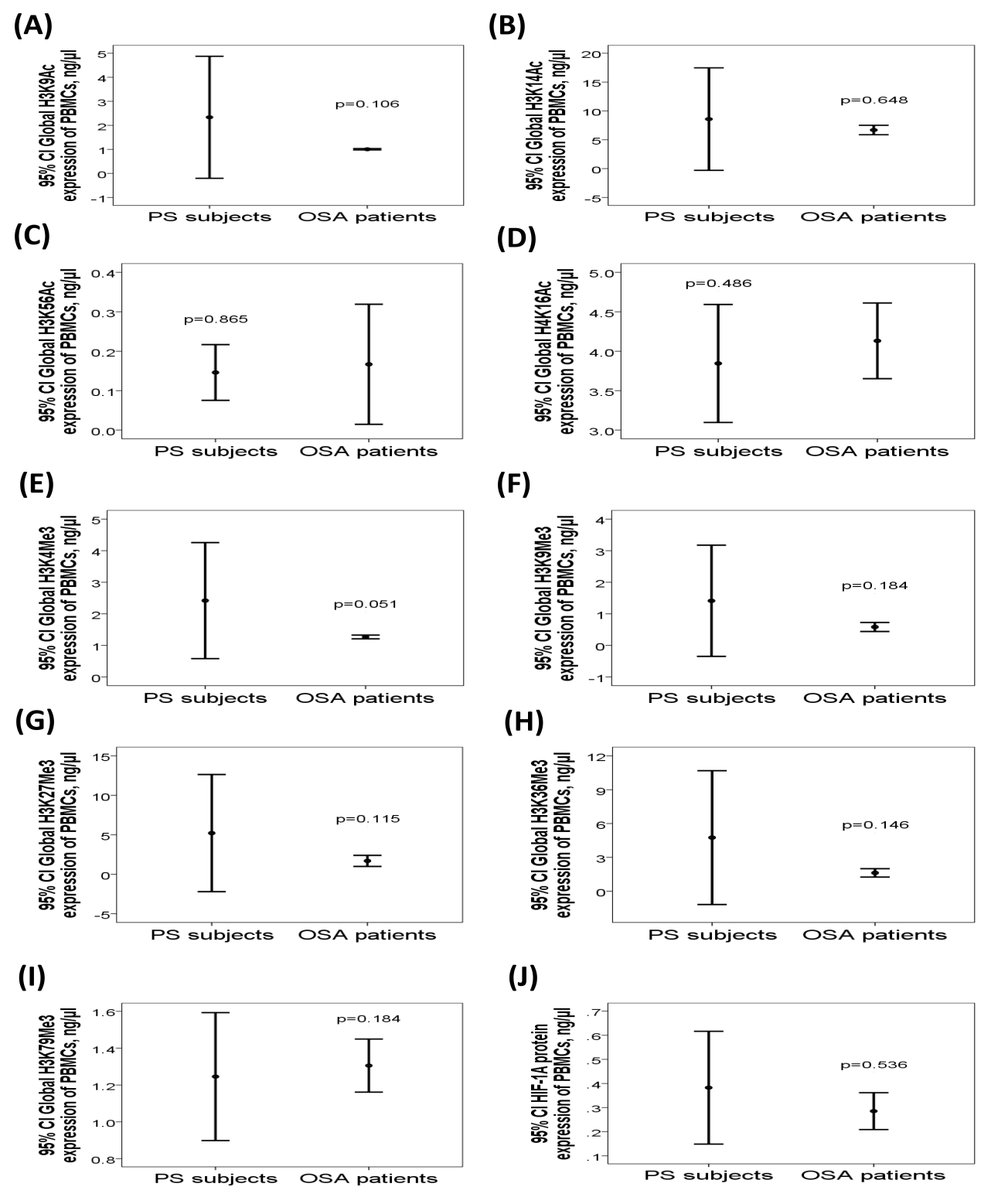
**

**Supplementary Figure S1. Histone modification patterns not related to obstructive sleep apnea (OSA).** There was no significant difference in global (A) H3K9Ac, (B) H3K14Ac, (C)　H3K56Ac, (D) H4K16Ac, (E) H3K4me3, (F) H3K9me3, (G) H3K27me3, (H) H3K36me3, (I) H3K79me3, or (J) HIF-1α protein expression between OSA patients and primary snoring (PS) subjects.

**
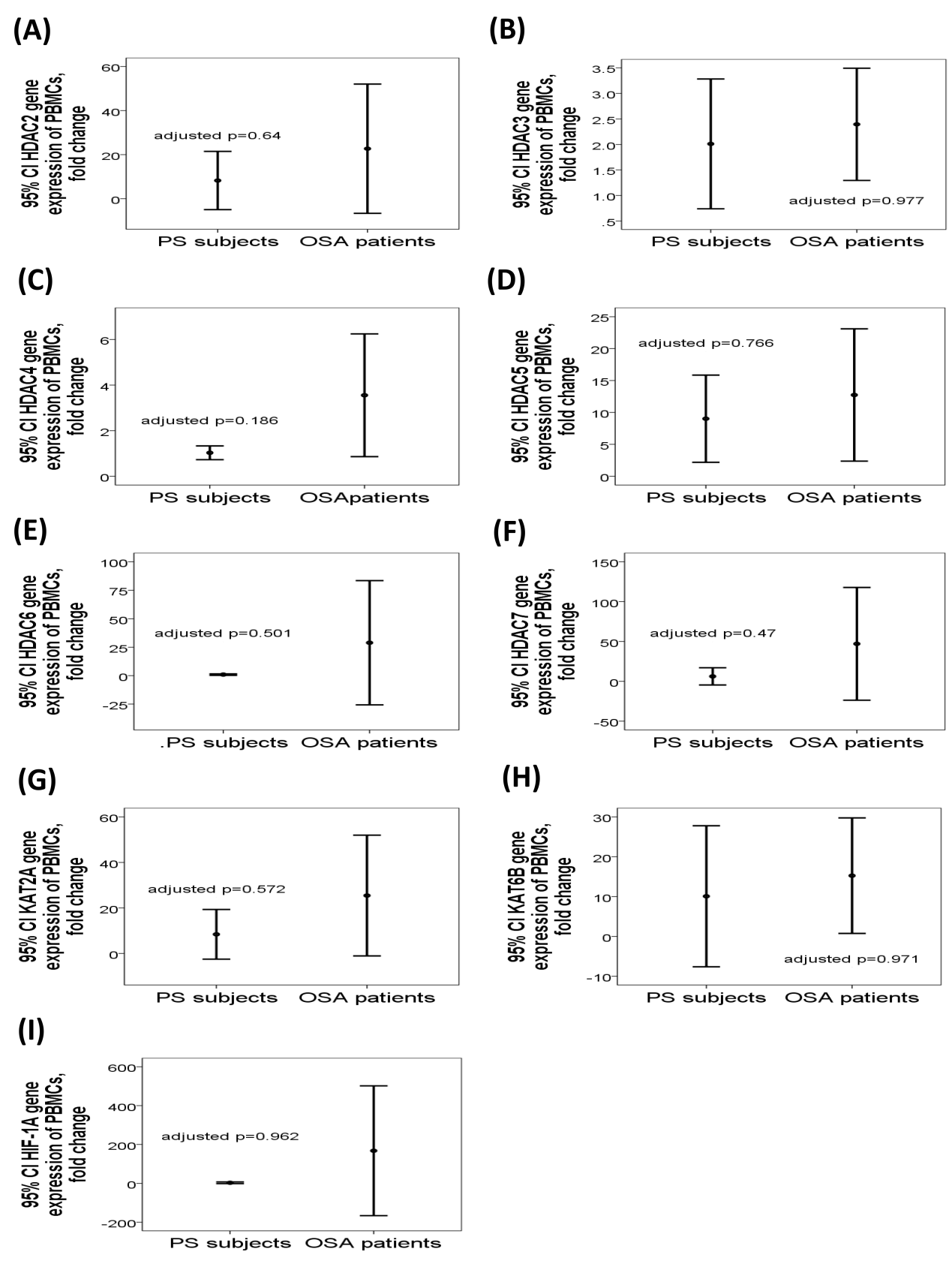
**

**Supplementary Figure S2. Histone acetylation modifying enzyme gene expressions not related to OSA.** There was no significant difference in gene expression level of the (A) *HDAC2*, (B) *HDAC3*, (C) *HDAC4*, (D) *HDAC5*, (E) *HDAC6*, (F) *HDAC7*, (G) *KAT2A*, (H) *KAT6B*, or (I) *HIF-1α* gene between OSA patients and PS subjects.

**
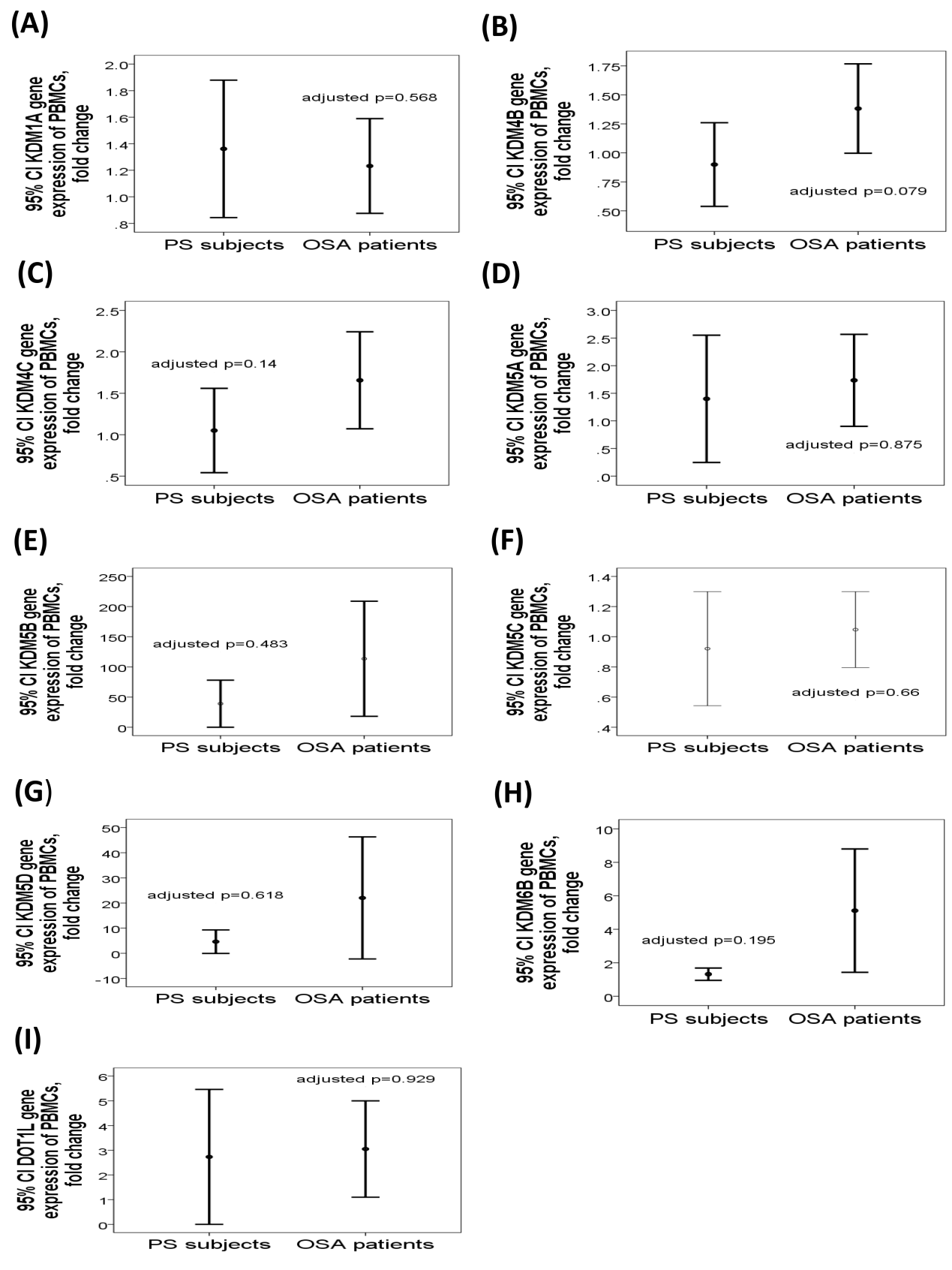
**

**Supplementary Figure S3. Histone methylation modifying enzyme gene expressions not related to OSA.** There was no significant difference in gene expression level of the (A) *KDM1A*, (B) *KDM4B*, (C) *KDM4C*, (D) *KDM5A*, (E) *KDM5B*, (F) *KDM5C*, (G) *KDM5D*, (H) *KDM6B*, or (I) *DOTL1* gene between OSA patients and PS subjects.

**
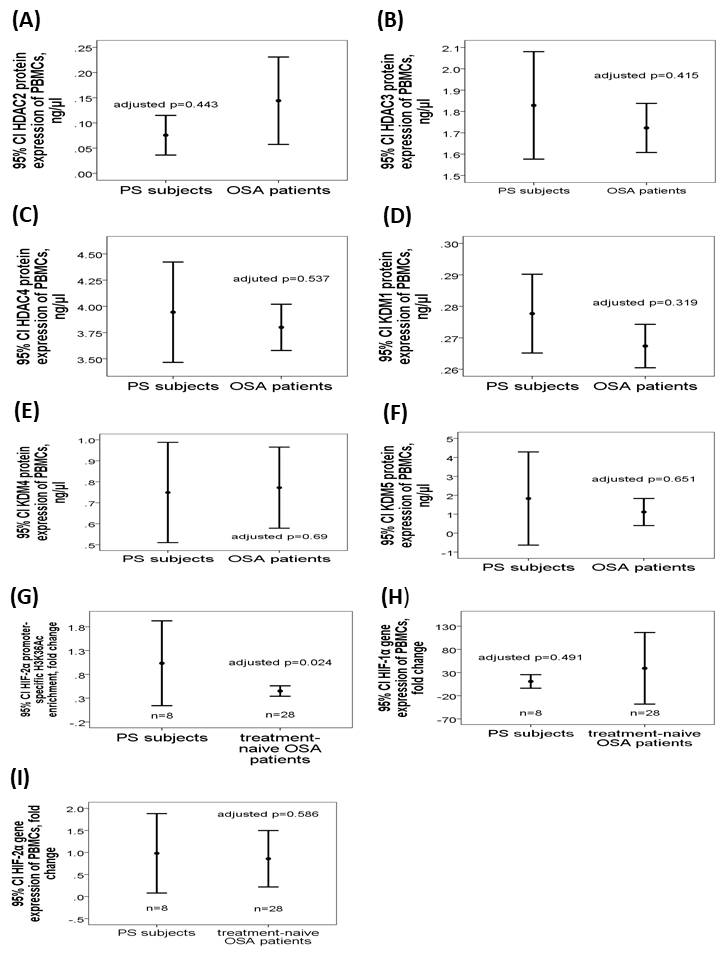
**

**Supplementary Figure S4. Histone modifying enzyme protein expression, *HIF-1α/HIF-2α* promoter-specific H3K36Ac, and *HIF-1α/HIF-2α* gene expression.** There was no significant difference in protein expression level of the (A) HDAC2, (B) HDAC3, (C) HDAC4, (D)KDM1, (E) KDM4, or (F) KDM5 between OSA patients and PS subjects of cohort 1. (G) HIF-2α gene promoter-specific H3K36Ac enrichment was decreased in OSA patients, but the difference was not significant in the non-parametric analysis. There was no difference in (H) *HIF-1α* or (I) *HIF-2α* gene expression level between OSA patients and PS subjects of cohort 2.

**Supplementary Table S1. Global H3K23Ac expression was negatively correlated with apnea hypopnea index (AHI) in multiple linear regression models.**

|  | Model 1: enter* | P value | Model 2: stepwise# | P value |
| --- | --- | --- | --- | --- |
|  | R square=0.183 | 0.293 | R square=0.165 | 0.015 |
|  | Regression coefficients |  | Regression coefficients |  |
| AHI | -0.004 | 0.046 | -0.004 | 0.043 |
| age | -0.008 | 0.25 |  |  |
| BMI | 0.021 | 0.37 |  |  |
| DM | -0.259 | 0.469 |  |  |
| HT | -0.208 | 0.168 | -0.271 | 0.032 |
| stroke | -0.077 | 0.822 |  |  |
| Heart disease | 0.014 | 0.95 |  |  |
| CKD | 0.295 | 0.645 |  |  |
| smoking | -0.038 | 0.781 |  |  |
| alcoholism | 0.1 | 0.578 |  |  |

BMI= body mass index; DM= diabetes mellitus; HT=hypertension; CKD= chronic kidney disease

***** Enter method means a procedure for variable selection in which all variables in a block are entered in a single step. Only when "enter method" is used, there will be p values for all variables

**#** Stepwise method: At each step, the independent variable not in the equation that has the smallest probability is entered, if that probability is sufficiently small. Variables already in the regression equation are removed if their probability becomes sufficiently large. The method terminates when no more variables are eligible for inclusion or removal.

**Supplementary Table S2. Global H3K36Ac expression was negatively correlated with apnea hypopnea index (AHI) in multiple linear regression models.**

|  | Model 1: enter | P value | Model 2: stepwise | P value |
| --- | --- | --- | --- | --- |
|  | R square=0.281 | 0.031 | R square=0.23 | <0.001 |
|  | Regression coefficients |  | Regression coefficients |  |
| AHI | -0.007 | 0.005 | -0.006 | 0.004 |
| age | -0.019 | 0.016 | -0.021 | 0.002 |
| BMI | 0.033 | 0.186 |  |  |
| DM | -0.035 | 0.927 |  |  |
| HT | -0.109 | 0.496 |  |  |
| stroke | -0.03 | 0.935 |  |  |
| Heart disease | 0.158 | 0.516 |  |  |
| CKD | 0.729 | 0.287 |  |  |
| smoking | 0.014 | 0.924 |  |  |
| alcoholism | -0.082 | 0.67 |  |  |

BMI= body mass index; DM= diabetes mellitus; HT=hypertension; CKD= chronic kidney disease

**Supplementary Table S3. Global H3K36Ac expression was negatively correlated with oxygen desaturation index (ODI) in multiple linear regression models.**

|  | Model 1: enter | P value | Model 2: stepwise | P value |
| --- | --- | --- | --- | --- |
|  | R square=0.235 | 0.1 | R square=0.201 | 0.002 |
|  | Regression coefficients |  | Regression coefficients |  |
| ODI | -0.005 | 0.042 | -0.005 | 0.026 |
| age | -0.02 | 0.016 | -0.022 | 0.002 |
| BMI | 0.035 | 0.184 |  |  |
| DM | -0.001 | 0.998 |  |  |
| HT | -0.1 | 0.547 |  |  |
| stroke | -0.05 | 0.894 |  |  |
| Heart disease | 0.161 | 0.523 |  |  |
| CKD | 0.782 | 0.268 |  |  |
| smoking | -0.014 | 0.923 |  |  |
| alcoholism | -0.129 | 0.514 |  |  |

BMI= body mass index; DM= diabetes mellitus; HT=hypertension; CKD= chronic kidney disease

**Supplementary Table S4. Global H3K36Ac expression was positively correlated with minimum oxygen desaturation (SaO2) in multiple linear regression models.**

|  | Model 1: enter | P value | Model 2: stepwise | P value |
| --- | --- | --- | --- | --- |
|  | R square=0.251 | 0.069 | R square=0.204 | <0.001 |
|  | Regression coefficients |  | Regression coefficients |  |
| Minimum SaO2 | 0.011 | 0.021 | 0.01 | 0.016 |
| age | -0.02 | 0.012 | -0.021 | 0.002 |
| BMI | 0.036 | 0.169 |  |  |
| DM | 0.068 | 0.86 |  |  |
| HT | -0.074 | 0.657 |  |  |
| stroke | -0.081 | 0.829 |  |  |
| Heart disease | 0.101 | 0.686 |  |  |
| CKD | 0.746 | 0.286 |  |  |
| smoking | -0.046 | 0.755 |  |  |
| alcoholism | -0.106 | 0.588 |  |  |

BMI= body mass index; DM= diabetes mellitus; HT=hypertension; CKD= chronic kidney disease

**Supplementary Table S5. H3K36Ac enrichment over the *HIF-1α* promoter region was negatively correlated with apnea hypopnea index (AHI) in multiple linear regression models.**

|  | Model 1: enter | P value | Model 2: stepwise | P value |
| --- | --- | --- | --- | --- |
|  | R square=0.494 | 0.005 | R square=0.35 | <0.001 |
|  | Regression coefficients |  | Regression coefficients |  |
| AHI | -0.024 | 0.004 | -0.016 | 0.01 |
| age | 0.008 | 0.869 |  |  |
| BMI | 0.099 | 0.06 |  |  |
| DM | -1.477 | 0.025 |  |  |
| HT | 0.569 | 0.314 |  |  |
| stroke | 3.462 | <0.001 | 2.718 | 0.001 |
| smoking | -0.169 | 0.533 |  |  |
| alcoholism | 0.269 | 0.781 |  |  |

BMI= body mass index; DM= diabetes mellitus; HT=hypertension

**Supplementary Table S6. H3K36Ac enrichment over the *HIF-1α* promoter region was negatively correlated with oxygen desaturation index (ODI) in multiple linear regression models.**

|  | Model 1: enter | P value | Model 2: stepwise | P value |
| --- | --- | --- | --- | --- |
|  | R square=0.661 | <0.001 | R square=0.505 | <0.001 |
|  | Regression coefficients |  | Regression coefficients |  |
| ODI | -0.026 | 0.001 | -0.023 | 0.001 |
| age | 0.01 | 0.584 |  |  |
| BMI | -0.039 | 0.587 |  |  |
| DM | -1.189 | 0.071 |  |  |
| HT | 1.128 | 0.054 |  |  |
| stroke | 4.892 | 0.001 | 4.6 | <0.001 |
| smoking | -0.015 | 0.957 |  |  |
| alcoholism | -0.122 | 0.921 |  |  |

BMI= body mass index; DM= diabetes mellitus; HT=hypertension

**Supplementary Table S7. H3K36Ac enrichment over the *HIF-1α* promoter region was positively correlated with minimum oxygen saturation (SaO2) in multiple linear regression models.**

|  | Model 1: enter | P value | Model 2: stepwise | P value |
| --- | --- | --- | --- | --- |
|  | R square=0.674 | <0.001 | R square=0.628 | <0.001 |
|  | Regression coefficients |  | Regression coefficients |  |
| Minimum SaO2 | 0.061 | 0.001 | 0.058 | <0.001 |
| age | 0.014 | 0.443 |  |  |
| BMI | -0.054 | 0.375 |  |  |
| DM | -1.109 | 0.084 | -1.433 | 0.004 |
| HT | 1.369 | 0.023 | 1.248 | 0.001 |
| stroke | 4.623 | <0.001 | 4.744 | <0.001 |
| smoking | 0.027 | 0.92 |  |  |
| alcoholism | -0.404 | 0.737 |  |  |

BMI= body mass index; DM= diabetes mellitus; HT=hypertension
